# Genome-Wide Mapping of Histone H3 Lysine 4 Trimethylation (H3K4me3) and Its Involvement in Fatty Acid Biosynthesis in Sunflower Developing Seeds

**DOI:** 10.3390/plants10040706

**Published:** 2021-04-06

**Authors:** Antonio J. Moreno-Pérez, José M. Santos-Pereira, Raquel Martins-Noguerol, Cristina DeAndrés-Gil, M. Adrián Troncoso-Ponce, Mónica Venegas-Calerón, Rosario Sánchez, Rafael Garcés, Joaquín J. Salas, Juan J. Tena, Enrique Martínez-Force

**Affiliations:** 1 Instituto de la Grasa (CSIC), Building 46, UPO Campus, Ctra. de Utrera km 1, 41013 Seville, Spain; ajmoreno@ig.csic.es (A.J.M.-P.); rmnoguerol@ig.csic.es (R.M.-N.); cdeandresgil@ig.csic.es (C.D.-G.); mvc@ig.csic.es (M.V.-C.); rsanchez@ig.csic.es (R.S.); rgarces@ig.csic.es (R.G.); jjsalas@ig.csic.es (J.J.S.); 2 Centre de Recherche Royallieu, Université de Technologie de Compiègne, Alliance Sorbonne Université, UPJV, UMR-CNRS 7025, Enzyme and Cell Engineering, 60200 Compiègne, France; adrian.troncoso-ponce@utc.fr; 3 Centro Andaluz de Biología del Desarrollo (CSIC-UPO), Building 20, UPO Campus, Ctra. de Utrera km 1, 41013 Seville, Spain; jjtenagu@upo.es (J.J.T.); jmsanper1@upo.es (J.M.S.-P.)

**Keywords:** histone modification, H3K4m3, ChIP-Seq analysis, sunflower, fatty acid biosynthesis

## Abstract

Histone modifications are of paramount importance during plant development. Investigating chromatin remodeling in developing oilseeds sheds light on the molecular mechanisms controlling fatty acid metabolism and facilitates the identification of new functional regions in oil crop genomes. The present study characterizes the epigenetic modifications H3K4me3 in relationship with the expression of fatty acid-related genes and transcription factors in developing sunflower seeds. Two master transcriptional regulators identified in this analysis, VIV1 (homologous to Arabidopsis ABI3) and FUS3, cooperate in the regulation of WRINKLED 1, a transcriptional factor regulating glycolysis, and fatty acid synthesis in developing oilseeds.

## 1. Introduction

Histone modifications play an integral role in plant development [1]. These epigenetic modifications regulate gene transcription through chromatin remodeling, since chromatin organization participates in the control of gene expression. These regulatory processes take place through conserved mechanisms in plants [2], including methylation, acetylation, phosphorylation, and ubiquitination of histones [3,4,5,6]. Moreover, the combination of different histone modification patterns finetunes gene expression [7,8,9]. A large number of biological processes have been demonstrated to be affected by histone modifications in plants, including cellular growth, light and temperature responses, flowering, hormone responses, and circadian regulation [10,11]. However, histone modifications have been poorly studied in the context of de novo fatty acid (FA) biosynthesis in oilseeds. In *Arabidopsis thaliana*, mutations affecting histone methylation or acetylation-related genes have resulted in modifications of FA content and composition within seeds [12,13], suggesting that epigenetic marks participate in the control of FA synthesis in developing seeds. Seed oils are constituted almost entirely of triacylglycerol (TAG) ester molecules, which contain FA as the most abundant form of reduced carbon chains [14]. The value and applications of seed oils are largely based on their FA composition [15]. Therefore, epigenetic mechanisms could contribute to obtaining tailored edible vegetable oils with improved nutritional properties [12] and oils with enhanced industrial applications.

Sunflower (*Helianthus annuus* L.) is an important oilseed crop cultivated worldwide. Like many other oilseed crops, several attempts to modify its oil composition have been made, for example, the development of new sunflower cultivars, obtained from mutagenized seeds, with increased level of saturated fatty acids [16,17,18,19]. The biochemical pathways leading to oil synthesis and accumulation in sunflower seeds have been largely studied (reviewed by the authors [20]). However, the regulation of these processes is only partially characterized and remains a challenge. In plants, de novo FA biosynthesis takes place within plastids of non-photosynthetic tissues and in the chloroplasts of the vegetative ones. In summary, this biosynthetic process involves the formation of acyl chains through successive cycles of 2-carbon additions to the acyl-acyl carrier protein (ACP) derivates until an 18-carbon saturated chain is reached [21]. The FA biosynthesis pathway is regulated at the transcriptional level [22], and certain genes encoding enzymes that participate in this pathway are coregulated by common regulatory elements activated by WRINKLED1 (WRI1) [23]. WRI 1 is a member of the APETALA2/ethylene-responsive element binding (AP2/EREBP) proteins family [24]. This transcription factor not only activates the expression of FA synthesis-related genes but also promotes the expression of several glycolytic enzymes directing the carbon flux toward the oil synthesis [25]. The function of this transcription factor is conserved in Arabidopsis, *Zea mays*, *B. napus,* and other species [26]. WRI1 has been considered the main transcriptional regulator of de novo FA biosynthesis. *WRI1* locus was first described by Focks and Benning [27] who demonstrated that reduced accumulation of oil in Arabidopsis *wri1* mutants, of 80% less than wild-type seeds, corresponded to the conversion of carbohydrates into precursors of fatty acids and adversely affected triacylglycerol. Thus, it constitutes a promising tool for increasing the oil yield in future transgenic varieties of traditional oil seed crops [28]. Despite its importance during oil biosynthesis and accumulation, its contribution to FA synthesis regulation is not completely understood in sunflower. In *Arabidopsis*, the expression of *WRI1* is regulated by other master transcription factors, LEC1, ABI3, FUS3, and LEC2 (named LAFL collectively), which regulate both the seed maturation and the accumulation of storage compounds [29,30,31,32]. Epigenetic modifications, including histone methylation, partially control the activation and repression of LAFL transcription factors [33,34].

In plants, lysine 4 trimethylation on histones H3 (H3K4me3) and acetylation of lysine 9 (H3K9Ac) at the transcription start site (TSS) are considered predictors of gene expression [35].

The main objective of the present study was the characterization of the epigenetic modifications H3K4me3 in relation to the regulation of fatty acid biosynthesis in developing sunflower seeds. Therefore, we investigated the role of the modified histone H3K4me3 during different stages of FA synthesis in developing sunflower seeds (13 days and 28 days after anthesis) using chromatin immunoprecipitation followed by high-throughput sequencing (ChIP-Seq) techniques. Consequences of epigenetic modifications upon the expression of enzymes and transcription factors involved in FA synthesis are discussed.

## 2. Materials and Methods

### 2.1. Biological Material and Growth Condition

Plants used in this work corresponded to the fixed high-oleic mutant line CAS-9 (Collection of Andalusian Sunflower from Instituto de la Grasa, CSIC, Seville, Spain). Plants were grown in growth chambers on a 25 °C/15 °C (day/night) cycle with a 16 h photoperiod and a photon flux density of 200 μmol m^−2^s^−1^. Developing seeds from sunflower plants were harvested at 13 days (early fatty acid biosynthesis stage), 18 days (intermediate fatty acid biosynthesis stage), and 28 days (late fatty acid biosynthesis stage) after anthesis (DAA) and frozen until use. For all experiments performed in this study, 13 DAA and 18 DAA samples were used, and the 18 DAA sample was used only in quantitative real-time polymerase chain reaction (RT-qPCR) experiments.

### 2.2. Isolation and Immunoprecipitation of Chromatin

Chromatin preparation was performed essentially as described by Sequeira-Mendes et al. [36] using 13 DAA and 28 DAA developing seeds. The seeds were fixed for 15 min in 1% formaldehyde (in 200 mM phosphate buffer) at room temperature, quenched for 5 min with 0.125 M glycine, washed in PBS, and frozen at −80 °C. Fixed seeds were homogenized in 2 mL sonication buffer (20 mM Tris-HCl pH 8.0, 70 mM KCl, 0.125% NP-40, 1 mM EDTA, 10% glycerol, and 1x Roche Complete protease inhibitors cocktail), with a Dounce Homogenizer on ice. Fixed seeds were then centrifuged for 5 min at 2300 g at 4 °C. Pelleted nuclei were resuspended in nuclear lysis buffer (50 mM Tris-HCl pH 7.5, 10 mM EDTA, 1% SDS, 1x Roche Complete protease inhibitors cocktail), kept on ice for 5 min, and diluted with ChIP dilution buffer (16.7 mM Tris-HCl pH 7.5, 1.2 mM EDTA, 167 mM NaCl, 0.01% SDS, 1.1% Triton-X100). Then, chromatin was sonicated in a Bioruptor UCD-200 (Diagenode; Denville, NJ, USA) sonicator (high intensity, 30 s ON, 30 s OFF for 15 min) and centrifuged for 5 min at 18,000 g at 4 °C. The recovered supernatant contained soluble chromatin fragments from 200 bp to 600 bp. Genomic fragments were measured in agarose gel by taking 20 μL of sonicated chromatin that was treated with 1 μL of RNase 10 mg/mL 30 min at 37 °C and 1 μL of proteinase K 10 μg/mL 1 h at 65 °C, purified by phenol-chloroform-isoamyl alcohol extraction and precipitated with 100% ethanol.

The rest of ChIP-Seq experiment was carried out as described previously by Santos-Pereira et al. [37] with minor modifications. First, 1 μg of the DNA/protein complex was inmunoprecipitated with anti-H3K4me3 antibody (Abcam Cat#ab8580). After cross-linked reversal and DNA purification, the ChIP DNA was quantified with the Qubit HS dsDNA kit (Thermo Fisher Scientific; Waltham, MA, USA). Illumina libraries for sequencing were generated, and the libraries were sequenced using the HiSEq 4000 Sequencing System (Illumina; San Diego, CA, USA) following the manufacturer’s instructions.

### 2.3. Computational Analysis of Sequencing Data

#### 2.3.1. Quality Analysis and Filtering

Preprocessing of the sequence files obtained from Illumina sequencing (FASTQ files) was carried out using the FASTX-Toolkit v0.0.13 [38] in the command-line version. The main tools used were the FASTQ Quality Filter and FASTQ Quality Trimmer, following the developer’s instructions. First, the sequences were trimmed, setting 20 as the quality threshold and establishing the minimum length as 30 nucleotides. Then, the sequences were filtered again using more restrictive quality criteria (minimum quality threshold = 20 for 90% of the bases).

#### 2.3.2. Mapping Sequences

Sequences were mapped using BWA-MEM-0.7.16a software [39] and the reference genome from sunflower inbred line XRQ [40]. Later, the mapping quality was analyzed with the samtools flagstat v1.10.2 tool [41].

#### 2.3.3. Aligned Sequences Evaluation and Peak-Calling

Duplicated reads were deleted using MarkDuplicates from Picard Tools v1.141 (http://broadinstitute.github.io/picard/, accessed date 19 March 2021) according to Yan et al. [42]. Then, quality metrics normalized strand cross-correlation (NSC), measured as the ratio of the maximal cross-correlation value divded by the background cross-correlation, and relative strand cross-correlation coefficient (RSC), defined as the result of the ratio of the fragment-length cross-correlation value minus the background cross-correlation value divided by the phantom-peak cross-correlation value minus the background cross-correlation value, were calculated using R script Phantompeakqualtools v1.2 [43]. Peak-calling was performed using MACS2-2.1.4 (Model-based Analysis of ChIP-Seq data) algorithm [44,45]. The reference genome was the same described in the previous section, and the *p*-value was 0.001.

#### 2.3.4. ChIP Peaks Annotation and Functional Enrichment Analysis

ChIP peaks annotation and visualization of ChIP peaks coverage over chromosomes and profiles of peaks binding to TSS regions were carried out using the R package ChIPseeker 1.22.0 version [46]. TSS regions, which are defined as the flanking sequence of the TSS sites, were established at −3000 and +3000 bases. The Gene Ontology (GO) annotation and the functional enrichment analysis was performed in PANTHER16.0 software [47,48] the sunflower genome described above as a reference.

#### 2.3.5. Motif Analysis: *WRI1* Binding Sequences

The Homer v4.10 tool [49] and *WRI1* DNA-binding site matrix were used to perform the motif analysis and the selection of sequences potentially regulated by *WRI1* following the pipeline proposal by the software developer.

### 2.4. mRNA Preparation and cDNA Synthesis

mRNA was isolates from 13 DAA, 18 DAA, and 28 DAA developing seeds and cDNA was synthetized as previously described by Moreno-Pérez et al. [50].

### 2.5. Quantitative Real-Time PCR

The expression levels of sunflower *WRI1* were analyzed by RT-qPCR using cDNAs generated from developing sunflower seeds (13, 18 and 28 DAA). The specific pair of primers used were qPCR_Hawri1_F (5′-TTAAGGCGAAGGAGCAGTGG-3′) and qPCR_Hawri1_R (5′-GCTTCCTCTTGAGTGCCGTA-3′). The qPCR reactions were performed using SsoAdvanced universal SYBR Green supermix (Bio-Rad, USA), and the PCR amplification consisted of an initial denaturation at 95 °C for 15 s, 39 cycles of 95 °C for 15 s, and 60 °C for 30 s. The expression of the actin gene HaAct1 (GenBank Accession FJ487620) was measured to normalize the data using the primers HaActin-qpcr-F4 (5′-GCTAACAGGAAAAGATGACT-3′) and HaActin-qpcr-R4 (5′-ACTGGCATAAAGAGAAAGCACG-3′). The 2^−ΔΔCt^ (Livak) method [51] was used to calculate the relative expression of the gene.

### 2.6. Analysis of Fatty Acid Composition

Three replicates of 200 mg of 13 DAA and 28 DAA developing seeds were used for total lipid extraction. The method used was the previously described by Hara and Radin [52] with some modifications. First, 2 mL isopropanol were added to the samples and heated at 80 °C for 20 min to improve the yield extraction. Accordingly, 3 mL hexane were added to reach a 3:2 hexane:isopropanol proportion (*v*/*v*). Then, 1.5 mL of 6.7% Na_2_SO_4_ (*w*/*v*) was added, and samples were mixed. The upper phase containing the lipids was transferred into clean tubes, and the remaining aqueous phase was re-extracted with 2.5 mL hexane:isopropanol 7:2 (*v*/*v*). The upper phase was recovered and combined with the previously extracted phase. The solvent was evaporated under an inert nitrogen atmosphere at 40 °C. The lipid extract was then trans-esterified to their fatty acids methyl esters (FAMEs) by methylation reaction performed heated at 80 °C for 1 h after the addition of 2 mL of methanol/toluene/sulfuric acid (88/10/2; *v*/*v*/*v*). Then, 2 mL heptane was added, and the upper phase containing the FAMEs was extracted. The solvent was again evaporated under a nitrogen atmosphere and the FAMEs were resuspended in 200 µL heptane. Accordingly, gas chromatography was performed using a Hewlett Packard 6890 gas chromatograph (Palo Alto, CA, USA) with a Supelco SP-2380 fused-silica capillary column (30 m length, 0.25 mm i.d., 0.20 mm film thickness; Supelco, Bellefonte, PA, USA). Then, the peaks were identified by comparison of retention times with those from commercial standards (Sigma-Aldrich, USA). Heptadecanoic acid (17:0) was used as internal standard for fatty acid quantification.

### 2.7. Statistical Analysis

Statistical analysis was performed using the IBM SPSS v. 24.0 program (IBM Corp., Armonk, NY, USA). Data were tested for normality using the Kolmogorov–Smirnov test and the homogeneity of variance was tested with the Levene test. Accordingly, data were analyzed by ANOVA. Significant differences were determined by the Student-Newman-Keuls (SNK) test.

## 3. Results and Discussion

### 3.1. Computational Analysis of Sequencing Data

In the present study, a genome-wide examination of H3K4me3 histone marks in developing sunflower (*Helianthus annuus* L.) seeds was performed using the ChIP-Seq approach. A computational pipeline was designed for the sequencing data analysis. Optimized chromatin immunoprecipitation protocol for oleaginous seeds [53] was evaluated due to the difficulties associated to the immunoprecipitation and DNA isolation in rich-oil tissues. Finally, we successfully performed a standard protocol used in *Arabidopsis* seedlings [36]. Through aligning ChIP-Seq filtered reads to the sunflower reference genome (https://www.heliagene.org/HanXRQ-SUNRISE/, accessed date 19 March 2021; [40]) using Burrows-Wheeler Aligner (BWA-MEM-0.7.16a) software [39], it was found that only 28.45% and 52.35% of the reads were mappable to the genome in the 13 DAA and 28 DAA samples, respectively. It is likely that these low percentages were due to the annotation level of the available genome. Nevertheless, the number of reads and quality alignment are compatible with other ChIP-Seq experiments [54].

In order to quantify the enrichment grade of the immunoprecipitation, two quality metrics that evaluate the signal-to-noise ratio in a ChIP-Seq experiment, NSC and RSC, were calculated [55]. Samples from the 13 DAA showed a NSC value of 1.4, while the value of 28 DAA samples was 3.3 (NSC values higher than 1.1 indicate a high enrichment) (Figure S1). RSC values higher than 1 were obtained in experiments with a high enrichment. Both samples, 13 DAA and 28 DAA, showed values higher than 1, 2.0, and 1.5, respectively. In summary, both experiments showed high enrichment grades according to Marinov et al. [43]. Model-based analysis of the ChIP-Seq data (MACS2) algorithm [44,45] was used to obtain the peaks. From the ChIP-Seq data, 123,464 and 165,079 histone modification peaks were obtained in 13 DAA and 28 DAA samples, respectively.

### 3.2. Distribution of H3K4m3 Histone Modification in the Sunflower Genome

**Figure 1:**
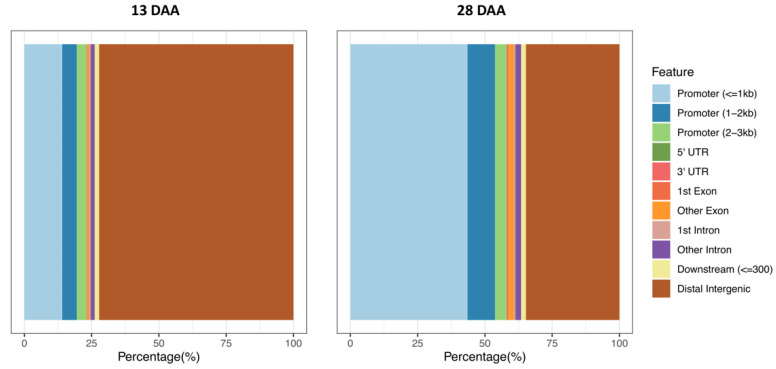
Genomic distribution of peaks identified in ChIP-Seq data. 13 DDA: Early stage of development. 28 DAA: Late stage of development.

The distribution of peaks identified in the ChIP-Seq data in the sunflower genome was characterized using the R package ChIPseeker 1.22.0 version [46]. The sunflower genome was classified into 11 classes between intergenic and genic regions (Figure 1). Unlike the 28 DAA sample, in the 13 DAA sample, H3K4me3 modifications were mainly distributed in distal intergenic regions. This deviation could be explained by the higher number of peaks detected in 28 DAA sample. Besides distal intergenic regions, the rest of classes were distributed quite similar in both samples (Figure 1). Promoter regions (<=1 kb, 1–2 kb, and 2–3 kb) were more commonly represented than the sum of other genic regions (5′ UTR, 3′ UTR, coding exon and intron). The distribution of H3K4me3 marks in sunflower histones differs from the pattern found in other species, such as *Oryza sativa* L. where UTRs (untranslated regions) and intron and exon regions were mainly represented [56]. In *Arabidopsis* and *Oryza sativa* L., H3K4m3 marks are located within genes and promoters, preferentially in genic regions 250–1000 bp downstream of the TSS [57,58,59,60]. Apart from distal intergenic regions, the sunflower distribution profile of the distance from peak to the TSS of the nearest gene were similar in both samples and comparable to other plant species (Figure S2).

### 3.3. Functional Enrichment Analysis

**Figure 2:**
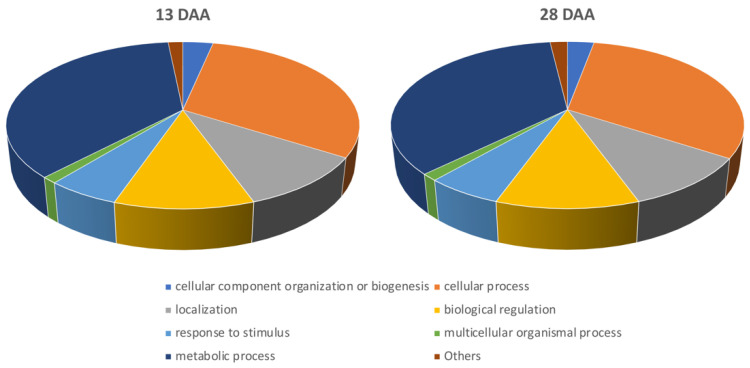
Gene Ontology annotation. Category: Biological Processes. 13 DDA: Early stage of development. 28 DAA: Late stage of development.

Through biological ontologies, functional enrichment analysis makes it possible to identify predominant biological themes from annotated genes using ChIP-Seq data [61]. Among genes annotated in the GO database, three major categories were obtained: Biological processes, cellular components, and molecular functions. In order to characterize de novo FA synthesis in developing sunflower seeds, this study focused mainly on the biological processes category (Figure 2). Both stages of seed development displayed a similar distribution of annotated genes within the different GO terms included in the previously mentioned category. The most represented biological processes sublevels were metabolic processes term (GO:0008152) (13 DAA: 36.2%; 28 DAA: 35.4%), followed by cellular process term (GO:0009987) (13 DAA: 30.6%; 28 DAA: 31.3%). Gene Ontology term enrichment analysis showed biological processes related to biosynthetic process (GO:0009058) for the upregulated genes only in earlier stage of seed development (fold enrichment: 1.16) (Figure S3).

Finally, exploring the latest term sublevel in the ontology, “fatty acid biosynthetic process” (GO:00066339), 21 genes involve in de novo fatty acid biosynthesis in sunflower were annotated in the 13 DAA sample and 30 genes in the 28 DAA sample.

### 3.4. Functional Annotation of Genes Involved in Fatty Acid Biosynthesis

Vegetable oils obtained from oilseeds are constituted mainly by triacylglycerols (TAG), which are the main storage lipid molecules and represent the chief source of energy and carbon for seedling development during germination before photosynthesis is established. Moreover, they constitute one of the major sources of calories in the human diet and an important renewable feedstock for industrial applications.

Although TAG assembly occurs outside plastids, fatty acid precursors are biosynthesized de novo within these organelles [28]. De novo fatty acid synthesis is a complex pathway which starts using acetyl-CoA as precursor. The route involves multiple reactions catalyzed by the fatty acid synthase complex (FAS) that produce the condensation of malonyl-ACP with acyl-ACP molecules [21]. When acyl-ACP molecules from 16- to 18-carbon atoms in length are generated, they are hydrolyzed into their acyl groups by the activity of acyl-ACP thioesterase enzymes and then exported to the cytosol [62]. These acyl-ACPs can be also desaturated by stearoyl-ACP desaturase (SAD) enzymes, producing palmitoleyl-ACP and oleyl-ACP.

**Figure 3:**
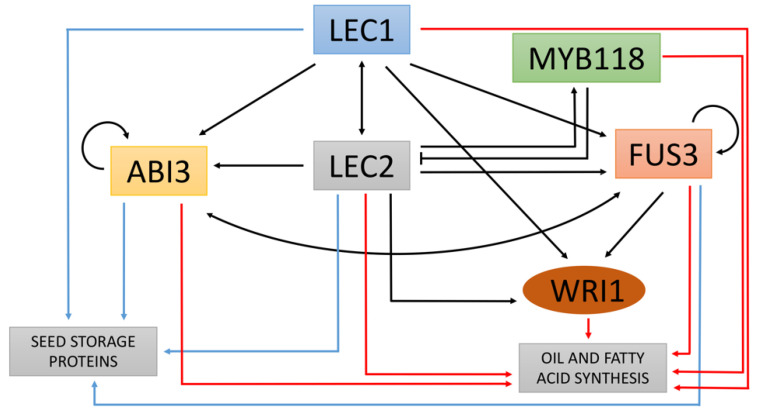
Schematic representation of the gene regulatory network controlling seed maturation. Arrows and blunted lines indicate activation and repression, respectively. Colors of the arrows stand for the regulation of transcription factors (black), seed storage proteins (blue) and genes involved in biosynthesis and storage of lipids (red). Modified from Fatihi et al., 2016.

In the model plant *Arabidopsis thaliana*, genetic and molecular analysis demonstrated the key role of LAFL (LEC1: LEAFY COTYLEDON1; ABI3: ABSCISIC ACID INSENSITIVE3, FUS3: FUSCA3; LEC2: LEAFY COTYLEDON2) transcription factors regulating seed maturation and the accumulation of storage compounds like reserve lipids [29,30,31,32]. LEC1 is a member of the NF-YB protein family, while ABI3, FUS3, and LEC2 belong to the family of B3 domain transcription factor (named AFL-B3 collectively). Homologous gene coding for LAFL transcription factors has been characterized in other plants [63,64,65] and similar regulation to *Arabidopsis* has been proposed. The regulatory interactions between the LAFL and their target genes regarding the control of storage compounds accumulation are summarized in the Figure 3 [66,67]. Target genes include seed storage proteins, enzymes involved in oil synthesis, and transcription factors such as MYB118, AGL12, and WRINKLED1 (WRI1) [68,69,70].

*Arabidopsis ABI3* and *FUS3* genes are marked by the activating H3K4m3 modification in seeds, indicating that their activation is at least partially regulated by chromatin-based mechanisms [33,34]. The current ChIP-Seq analysis points to a similar epigenetic control in developing sunflower seeds. Homologous genes coding for ABI3 and FUS3 transcription factors were detected using the BLAST+ v2.11.0 tool [71] in sunflower genome. *VIV1* and *FUS3* (*ABI3* and F*US3* homologous genes, respectively) were annotated in the ChIP-Seq experiment in 13 DAA and 28 DAA samples, indicating that they are in transcriptionally active regions marked by H3K4m3. Their fold enrichment in the ChIP-Seq experiment is shown in Table 1. These epigenetic similarities shared by Arabidopsis and sunflower strengthen the possibility of a common regulatory mechanism in both species. Moreover, based on the fold enrichments values found, this regulatory mechanism operates throughout different development stages in sunflower seeds. Moreover, annotation analysis of sunflower genes involved in FA biosynthesis was carried out. All genes annotated were located in the transcriptionally active regions marked by H3K4m3 (Table 2). In both stages, at least one enzyme encoding gene out of several isoforms catalyzing each reaction of the intraplastidial biosynthetic process was represented. These results corroborate the presence of active FA synthesis at 13 DAA and 28 DAA and are in consonance with the kinetics of oil accumulation in sunflower seeds previously described by Martínez-Force et al. [72].

### 3.5. Fatty Acid Composition in Developing Sunflower Seeds

In order to provide a better understanding between the functional annotation of genes involved in FA biosynthesis in both 13 DAA and 28 DAA stages and their oil composition, the lipid profile of both stages was analyzed. As expected, total lipid content of the later stage was significantly higher (Table 3). During sunflower seed formation, an active period of lipid synthesis occurs between 12–18 DAA. However, the main oil accumulation is produced between 18–19 DAA, and the highest lipid content is detected in seed from 20–25 DAA [72].

**Figure 4:**
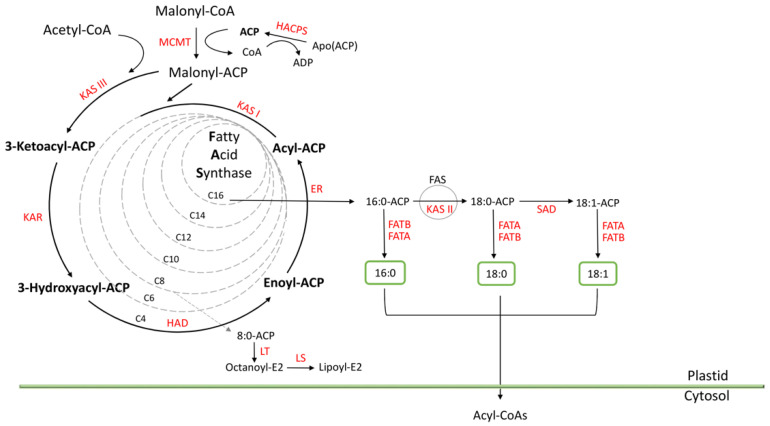
Scheme of intraplastidial fatty acid biosynthesis and export. MCMT: (Malonyl-CoA: ACP Malonyltransferase; KAS I: Ketoacyl-ACP Synthase I; KAS II: Ketoacyl-ACP Synthase II; KAS III: Ketoacyl-ACP Synthase III; KAR: Ketoacyl-ACP Reductase; HAD: Hydroxyacyl-ACP Dehydrase; ER: Enoyl-ACP Reductase; LT: Lipoyl Transferase; LS: Lipoyl Synthase; HACPS: Holo-ACP Synthase; SAD: Stearoyl-ACP Desaturase; FATA: Acyl-ACP Thioesterase A; FATB: Acyl-ACP Thioesterase B.

Regarding the qualitative composition, the FA detected in major proportion in both stages was unsaturated oleic acid (18:1^Δ9^) followed by linoleic acid (18:2^Δ9Δ12^). This profile is quite similar to that previously described by Martínez-Force et al. [73], although the ratio of unsaturated FA is different. The conversion from 18:0 to 18:1^Δ9^ was produced by SAD activity. Small differences in plant growth temperatures modify SAD activity [74] and, therefore, the unsaturated fatty acid ratio. Table 3 shows the different fatty acids experimentally detected. The saturated fatty acids were palmitic (16:0), stearic (18:0), and arachidic (20:0). Only the palmitic and stearic acids species displayed significantly differences (*p* < 0.05) between 13 DAA and 28 DAA samples. In the case of oleic and linoleic acid levels, no statistical differences were found between both stages. In the previous functional annotation of genes involved in FA biosynthesis (Table 2), as described above, at least one transcriptionally active gene copy of each enzyme that participates in the de novo fatty acid synthesis pathway was detected (Figure 4). Therefore, seeds from both stages present a fully FA synthesis machinery, showing a very similar composition in both stages.

### 3.6. WRI1 Gene Expression in Developing Sunflower Seeds

**Figure 5:**
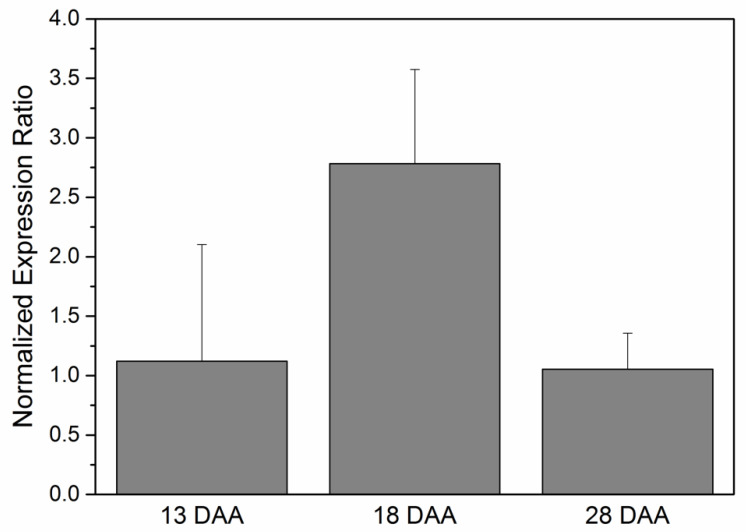
Normalized expression ratio (2^dCt) of sunflower *WRI1* in developing seeds determined by quantitative real-time polymerase chain (RT-qPCR) using actin gene from Helianthus annuus (GenBank FJ487620) as housekeeping. The data correspond to the mean ± SD of three independent samples.

*WRI1* belongs to the APETALA 2 transcription factor family and is a regulator of plant oil biosynthesis [25]. Although *WRI1* has been described and functionally characterized in different plants, including *Brassica napus*, *Camelina sativa*, *Persea americana*, *Ricinus communis*, and *Jatropha curcas* [75,76,77,78,79], among other species, the function of *WRI1* in sunflower seeds is not well characterized. *WRI1* is regulated, as described above, by LAFL transcription factors. Hence, *WRI1* is indirectly regulated by H3K4m3 chromatin modifications. *Arabidopsis WRI1* regulates the expression of genes involved in FA synthesis such as *KASI* (Ketoacyl-ACP Synthase I) or *MOD1* (Enoyl-ACP Reductase) [26]. Taking advantage of the H3K4m3 ChIP-Seq analysis in the present study (Table 2), *WRI1* regulated-genes from that list were selected. This selection was aimed at seeking *WRI*-binding motifs ([CnTnG](n)_7_[CG]; Figure S4; [80]) in the promoter region of annotated genes. According to the bioinformatic analysis, *KASI* (Ketoacyl-ACP Synthase I; HanXRQChr17g0564321) and *FABG* (Ketoacyl-ACP Reductase; HanXRQChr17g0536131) are putatively regulated by *WRI1* and, at the same time, under epigenetic H3K4m3 control. This result confirms the regulation previously described in *Arabidopsis* of *KASI* by *WRI1* and highlights a new potential regulation of *FABG* by *WRI1* in sunflower seeds. Moreover, the expression levels of sunflower *WRI1* were analyzed by RT-qPCR in different stages of sunflower seed development (13 DAA, 18 DAA, and 28 DAA). This analysis was performed in order to confirm the *WRI1* expression throughout the active oil synthesis stages. The highest *WRI1* transcript accumulation was observed at 18 DAA (intermediate stage of development) (Figure 5). This expression profile is different from that previously described in other plant seeds. For example, *Jatropha curcas* seeds showed higher expression during early stages of development but expression decreased by half in later stages [81]. Conversely, cottonseeds showed an opposite profile, with the highest expression being identified in medium-late stages [82].

Oilseeds FA biosynthesis-related genes displayed a specific expression profile where the maximum expression was reached in the intermediate stage of development [83]. This pattern is similar to the one observed for *WRI1* gene in developing sunflower seeds (Figure 5). Moreover, the peak of its expression corresponded with the highest rate of oil accumulation in sunflower seeds at 18-19 DAA [72]. The specific bell-shaped pattern of expression has been shown in a number of genes encoding core FA synthesis enzymes [83].

## 4. Conclusions

This study used chromatin immunoprecipitation followed by high-throughput sequencing (ChIP-Seq) to investigate the genome-wide distribution of the histone modification H3K4m3 in developing sunflower seeds. Although previous studies have described the difficulties associated with performing ChIP-Seq experiments in rich-oil seeds, a standard procedure for plants was carried out successfully in this case. The histone marks were enriched around the TSSs, corroborating that the distribution of histone modifications was associated with active transcription. GO analysis of annotated genes from ChIP-Seq experiments showed similar profiles during seed development, highlighting the similarities in terms of H3K4m3 marks during the period of active oil synthesis.

Moreover, the activation of ABI3 and FUS3 by H3K4m3 marks, previously described in *Arabidopsis*, was also identified in sunflower seeds, corroborating the role played by these epigenetic marks during seed maturation. In addition, genes encoding enzymes catalyzing every reaction of the FA synthesis pathway were represented in the functional annotation generated. Therefore, complete biosynthetic routes were found in both analyzed stages. The presence of an active pathway operating in both stages is in line with the oil accumulation kinetics in developing sunflower seeds. Furthermore, the expression of WRI1 correlates with the oil accumulation, consequently pointing to the role played by this transcription factor as an activator of the oil synthesis.

## Figures and Tables

**Table 1:** *ABI3* and *FUS3* homologous genes in sunflower and fold enrichment in ChIP-Seq experiment.

Arabidopsis		Sunflower		Fold Enrichment *	
Gene ID	Gene Name	Gene ID	Gene Name	13 DAF	28 DAF
AT3G24650	*ABI3*	HanXRQChr15g0468411	*VIV1*	6.37	5.39
AT3g26790	*FUS3*	HanXRQChr15g0475761	*FUS3*	5.9	6.32

* Measurement of overall enrichment for the region during peak-calling.

**Table 2:** Selected sunflower genes in ChIP-Seq data involved in intraplastidial fatty acid biosynthesis.

			ChIP-Seq Annotation	
Enzyme Activity	Arabidopsis Gene	Sunflower Homologous Gene	13 DAA	28 DAA
LS (Lipoyl Synthase)	AT5G08415.1	HanXRQChr05g0142151	Yes	Yes
		HanXRQChr12g0358451	Yes	Yes
		HanXRQChr17g0559041	Yes	Yes
LT (Lipoyl Transferase)	AT4G31050.1	HanXRQChr09g0268371	Yes	Yes
		HanXRQChr05g0141551	No	Yes
		HanXRQChr02g0056731	No	Yes
MCMT (Malonyl-CoA: ACP Malonyltransferase)	AT2G30200.1	HanXRQChr01g0012161	No	Yes
		HanXRQChr01g0012131	Yes	Yes
KASI (Ketoacyl-ACP Synthase I)	At5g46290	HanXRQChr17g0564321	Yes	Yes
		HanXRQChr04g0118391	Yes	Yes
		HanXRQChr13g0415471	Yes	Yes
		HanXRQChr17g0535001	No	Yes
		HanXRQChr01g0020441	No	Yes
		HanXRQChr01g0019841	No	Yes
KASII (Ketoacyl-ACP Synthase II)	At1g74960	HanXRQChr09g0237851	Yes	Yes
		HanXRQChr15g0497191	Yes	Yes
		HanXRQChr06g0178221	No	No
		HanXRQChr16g0508321	Yes	Yes
KASIII (Ketoacyl-ACP Synthase III)	At1g62640	HanXRQChr02g0049441	Yes	Yes
		HanXRQChr05g0148701	Yes	Yes
		HanXRQChr17g0550351	Yes	Yes
KAR (Ketoacyl-ACP Reductase)	At1g24360			
	At1g62610			
	At3g46170	HanXRQChr17g0536131	Yes	Yes
	At3g55290	HanXRQChr10g0318431	Yes	Yes
	At3g55310			
HAD (Hydroxyacyl-ACP Dehydrase)	At2g22230	HanXRQChr03g0066951	Yes	Yes
	At5g10160			
ER (Enoyl-ACP Reductase)	At2g05990	HanXRQChr02g0056711	No	Yes
		HanXRQChr17g0564011	Yes	Yes
HACPS (Holo-ACP Synthase)	At3g11470	HanXRQChr08g0212801	Yes	Yes
		HanXRQChr07g0194021	No	Yes
SAD (Stearoyl-ACP Desaturase)	At1g43800	HanXRQChr01g0023271		
	At2g43710			
	At3g02610			
	At3g02620		Yes	Yes
	At3g02630	HanXRQChr11g0343101	Yes	Yes
	At5g16230			
	At5g16240			
FATA (Acyl-ACP Thioesterase A)	At3g25110	HanXRQChr01g0015251	Yes	Yes
FATB (Acyl-ACP Thioesterase B)	At1g08510	HanXRQChr09g0240511	Yes	Yes
		HanXRQChr06g0180041	Yes	Yes
		HanXRQChr05g0138201	No	Yes
		HanXRQChr10g0311291	Yes	Yes
		HanXRQChr17g0553141	No	Yes
		HanXRQChr10g0311351	No	No
		HanXRQChr10g0311341	No	No
		HanXRQChr10g0311311	No	No
		HanXRQChr10g0311301	No	No
		HanXRQChr01g0013271	Yes	Yes
		HanXRQChr03g0062101	No	Yes
		HanXRQChr07g0197741	No	No

**Table 3:** Fatty acids composition (mol %) of developing sunflower seeds 13 days and 28 days after anthesis (DAA). Fatty acids: 16:0, palmitic acid; 18:0, stearic acid; 18:1^Δ9^, oleic acid; 18:2^Δ9Δ12^, linoleic acid; 20:0, arachidic acid. Data represent mean and standard deviation of three independent samples. * indicate significant differences (*p* ≤ 0.05).

	13 DAA	28 DAA
16:0	12.18 ± 2.64	4.74 ± 0.25 *
18:0	7.57 ± 0.56	2.83 ± 0.53 *
18:1^Δ9^	53.65 ± 13.23	71.92 ± 1.27
18:2^Δ9Δ12^	25.72 ± 10.01	20.13 ± 1.31
20:0	0.55 ± 0.08	0.13 ± 0.01 *
mg fatty acids/mg seed	0.020 ± 0.003	0.125 ± 0.007 *

## Data Availability

The data discussed in this publication have been deposited in NCBI’s Gene Expression Omnibus and are accessible through GEO Series accession number GSE171309 (https://www.ncbi.nlm.nih.gov/geo/query/acc.cgi?acc=GSE171309).

## Supplementary Materials

The following are available online at https://www.mdpi.com/article/10.3390/plants10040706/s1, Figure S1: Cross-correlation plot. Values of the quality metrics, NSC and RSC and Qtag. 13 DDA: early stage of development. 28 DAA: late stage of development. Figure S2: Distribution of H3K4m3 mark-binding loci relative to TSS. 13 DDA: early stage of development. 28 DAA: late stage of development. Figure S3: GO enrichment analysis. GO term: Biological process. A. 13 DAA sample. B. 28 DAA sample. Test type: Fisher’s Exact; Correction: False Discovery Rate (FDR). Barplot displaying only results for FDR *p* < 0.05. Figure S4. WRI1 DNA-binding site matrix.
