# Supplementary material for: Genome-Wide Mapping of Histone H3 Lysine 4 Trimethylation (H3K4me3) and Its Involvement in Fatty Acid Biosynthesis in Sunflower Developing Seeds
Source: Plants (Basel). 2021 Apr 6;10(4):706. doi: 10.3390/plants10040706 (PMC8067477; doi:10.3390/plants10040706)
Supplement: Supplementary file 1 [file plants-10-00706-s001.pdf]

Supplementary

Figure S1: Cross-correlation plot. Values of the quality metrics, NSC and RSC and Qtag. 13 DDA: early stage of development. 28 DAA: late stage of development.

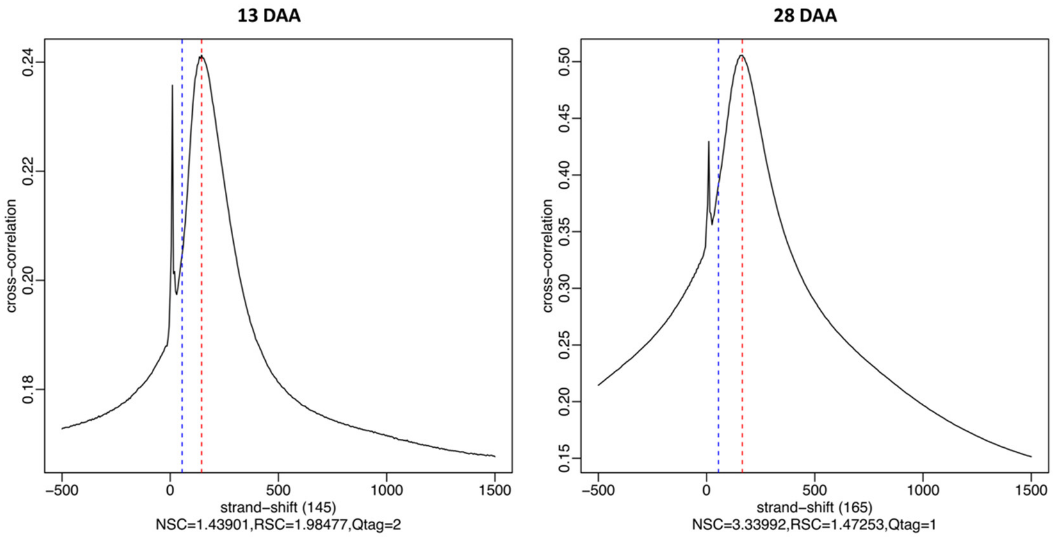

Figure S2: Distribution of H3K4m3 mark-binding loci relative to TSS. 13 DDA: early stage of development. 28 DAA: late stage of development.

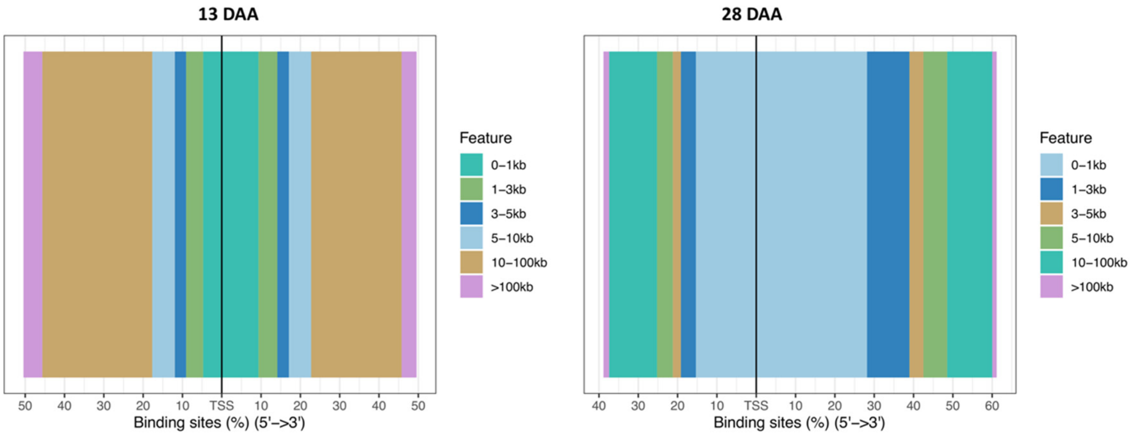

Figure S3: GO enrichment analysis. GO term: Biological process. A. 13 DAA sample. B. 28 DAA sample. Test type: Fisher's Exact; Correction: False Discovery Rate (FDR). Barplot displaying only results for FDR p<0.05.

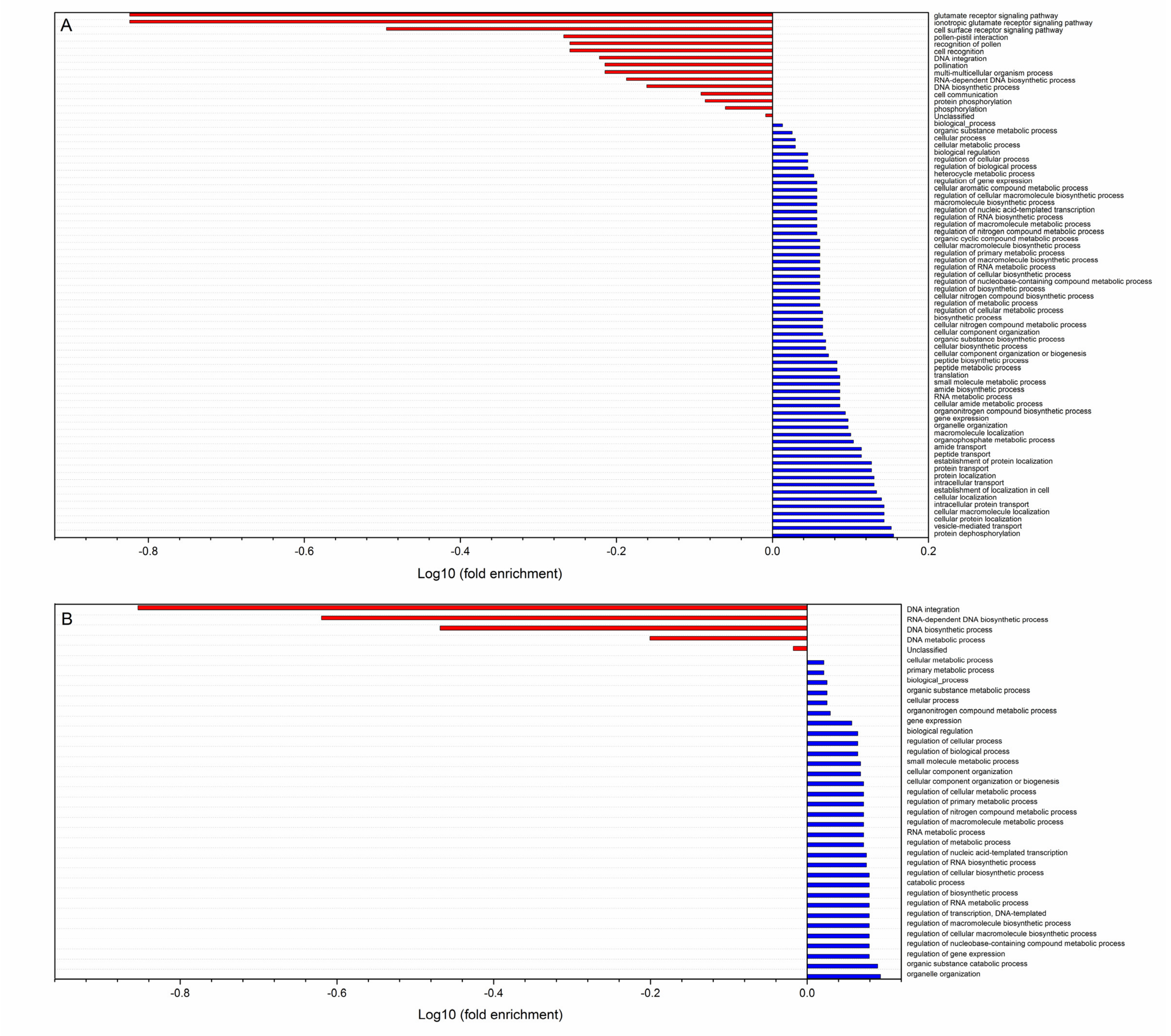

Figure S4. WRI1 DNA-binding site matrix.

Name: WRI1

Species: Arabidopsis thaliana

Family: AP2/EREBP

Logo:

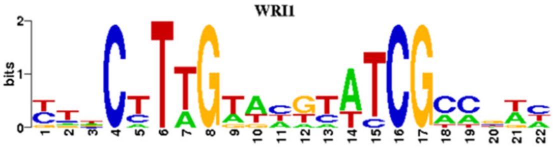

Matrix:

A	0	1	2	0	1	0	2	0	2	4	2	1	1	5	0	0	0	2	2	1	3	2
C	3	1	1	7	2	0	0	0	0	0	3	0	2	0	1	7	0	4	4	2	0	3
G	1	1	1	0	0	0	0	7	1	1	0	4	0	0	0	0	7	0	0	3	1	0
T	3	4	3	0	4	7	5	0	4	2	2	2	4	2	6	0	0	1	1	1	3	2

Max. score: 19.35

Threshold: 6.03
